# Therapeutic effects of intermittent fasting on high-fat, high-fructose diet; involvement of jejunal aquaporin 1, 3, and 7

**DOI:** 10.1016/j.heliyon.2024.e28436

**Published:** 2024-03-20

**Authors:** Heba M. Elhessy, Mohamed Berika, Yassmin G. Salem, Manal M. El-Desoky, Mamdouh Eldesoqui, Nora Mostafa, Ola A. Habotta, Nermeen H. Lashine

**Affiliations:** a Department of Anatomy and Embryology, Faculty of Medicine, Mansoura University, Mansoura, 35516, Egypt; b Department of Anatomy and Embryology, Faculty of Medicine, New Mansoura University, Mansoura, Egypt; c Department of Rehabilitation Science, College of Applied Medical Sciences, King Saud University, Saudi Arabia; d Department of Chemistry, Faculty of Medicine, Mansoura University, Mansoura, 35516, Egypt; e Department of Basic Medical Sciences, College of Medicine, AlMaarefa University, Diriyah, 13713, Riyadh, Saudi Arabia; f Department of Forensic Medicine and Toxicology, Faculty of Veterinary Medicine, Mansoura University, Mansoura, 35516, Egypt

**Keywords:** High-fat diet, Intermittent fasting, Aquaporin 1, Aquaporin 3, Aquaporin 7, Jejunum

## Abstract

**Background:**

Aquaporins (AQPs) are transmembrane channel proteins. Aquaporin 1 (AQP1), Aquaporin 3 (AQP3), and Aquaporin 7 (AQP7) are expressed in the jejunum. The purpose of this study was to ascertain how a high-fat high-fructose diet (HFFD) and intermittent fasting (IF) affect AQP1, AQP3, and AQP7 expression in the rat jejunum.

**Methods:**

Sixteen adult male rats were divided into control rats (n = 4) fed on a basal diet and water ad libitum for 12 weeks; IF control rats (n = 4) followed the IF protocol, HFFD-fed rats (n = 8) fed HFFD for eight weeks, and rats were randomized into two groups: HFFD only or HFFD and IF protocol from the beginning of the 9th week until the end of the experiment. The lipid profile values were assessed after 12 weeks. Jejunal oxidative markers (malondialdehyde and reduced glutathione) and AQP1, AQP3, and AQP7 mRNA expression were measured. Jejunal sections were used for morphometric analysis of villus length and crypt depth. Immunohistochemical evaluation of AQP1, AQP3, and AQP7 expression was also performed.

**Results:**

IF ameliorates HFFD-induced lipid profile, oxidative stress, and jejunal morphometric changes. The results of both mRNA expression using PCR and immunohistochemistry showed a significant increase in AQP1, AQP3, and AQP7 expression in HFFD, whereas IF caused a decline in this expression.

**Conclusion:**

These findings suggest that IF can reduce inflammation, and oxidative stress and restore jejunal morphology caused by HFFD.

## Highlights

• High Fat High fructose diet causes Jejunal histopathological changes and Jejunal oxidative stress.
• Intermittent Fasting ameliorates the deleterious effect of the high fat on Jejunum.
• Aquaporin1, Aquaporin3, and Aquaporin 7 expression increase in high-fat high fructose diet.
• Intermittent fasting is an effective anti-obesity strategy.
• Aquaporin1, Aquaporin3, and Aquaporin 7 can be used as therapeutic targets.

## 1 Introduction

Numerous metabolic issues, such as hyperlipidemia, insulin resistance, type 2 diabetes mellitus (T2DM), non-alcoholic fatty liver disease (NAFLD), and metabolic syndrome, have long been associated with an unhealthy diet, particularly high-fat diet (HFD) or “Western” diet [1]**.** It is well-accepted that a high-fat diet has a significant negative influence on the brain, behavior, and cognition [2]. Quality of life is affected by these well-known metabolic disorders, which ultimately cause premature death globally [3]. Rats have been fed fat-enriched diets for decades to imitate obesity, dyslipidemia, and insulin resistance [4,5]**.**

The most significant barrier separating a person from their environment is the gastrointestinal tract. The intestinal tract is responsible for absorbing essential nutrients from food and safeguarding against a range of poisons and bacteria. The intestinal barrier system, which consists of a mucus layer, intestinal epithelial cells, tight junctions, immune cells, and gut bacteria, is vulnerable to external influences such as dietary fats [6]**.** Consumption of meals high in fructose and saturated fat may disrupt this barrier [7]**.** Increased intestinal permeability affects the gut by increasing barrier-disrupting cytokines such as tumor necrosis factor (TNF), interleukin (IL) 1 B, IL6, and interferon (IFN), and decreasing barrier-forming cytokines such as IL10, IL17, and IL22. Overall, HFD adversely alters intestinal mucus composition and enriches gut microbiota with species that disturb barrier function [6]**.** This process eventually results in metabolic endotoxemia [8]and local intestinal inflammation [9]**.**

In the normal digestion process, dietary triglycerides (TGs) are broken down by lipases in the small intestine and transformed into mono- and diacylglycerols, which are subsequently absorbed by the intestinal mucosa. Monoacylglycerols and free fatty acids are converted into TGs in enterocytes and released through their basolateral membrane into the lymphatic system as low-density lipoproteins called chylomicrons. Chylomicrons exit the lymph into the bloodstream and circulate until they reach adipose, cardiac, and skeletal muscle tissues [10]**.** Aquaglyceroporins enhance glycerol permeability through membranes; hence, it is essential to regulate glycerol transport through aquaporins (AQPs) to regulate fat synthesis, lipolysis, gluconeogenesis, and energy homeostasis [11]**.**

The Aquaporins (AQPs) are integral membrane proteins. They serve the purpose of moving tiny molecules such as H2O2, glycerol, ammonia, urea, water, and other substances through biological membranes [12]. aquaporins in mammals are split into three typical classes [13].

I. The ‘classical’ aquaporins, AQP0, AQP1, AQP2, AQP4, and AQP5, AQP6, AQP8, are water-selective proteins.
II. Aqua-glyceroporins, also known as glycerol facilitation-like proteins (GLP), such as AQP3, AQP7, AQP9, and AQP10, are homologs that are permeable to both water and glycerol.
III. SuperAQPs, which are composed of AQP11 and AQP12, have limited sequence homology with other AQP groups.

Aquaporin 1 was evaluated because it is expressed throughout the digestive tract at sites that are involved in fat digestion and absorption. Aquaporin 1 is expressed in the central lacteal cells of the small intestine, where it facilitates chylomicron production [14]**.** Studies confirmed the role of AQP1 in fat digestion by demonstrating intestinal dietary fat misprocessing in AQP1 null animals [15,16]**.** AQP7 is involved in glycerol absorption from dietary fat, as it is present in the small intestine apical membrane enterocytes and exits through AQP3 located in the basolateral membrane of these enterocytes [10]**.** AQP3 has also been shown to be responsible for glycerol permeability in the kidneys [17]**.**

Intermittent fasting (IF) can lower fat mass, insulin, and glucose levels in the blood, as well as the probability of acquiring age-related diseases. IF promotes adaptive cellular responses that have been preserved throughout evolution, including stress response, autophagy, and mitochondrial function. Additionally, it modifies the circadian rhythms of several hormones, including insulin and leptin, whose levels fluctuate according to the abundance and scarcity of food [18]**.** Apart from its general impacts, IF induces tissue-specific metabolic alterations, including a reduction in liver fat content, remodeling of adipose tissue, correction of obesity-related problems, and a gain in lean mass [[19], [20], [21]]**.**

Considering the presumed beneficial effects of IF on HFD-induced pathology in multiple organs, this study is designed to focus on its possible ameliorative effects on the intestine and to estimate the expression AQPs involved in fat absorption (AQP1, AQP3, and AQP7) to check the possibility of using them as a target for controlling intestinal fat absorption or as a potential therapeutic target through both biochemical and histopathological investigations. The jejunal part of the intestine was selected because it has been noted that dietary fat has the greatest effects on gene expression in the proximal and middle portions of the small intestine, where fat absorption often occurs [22] and AQP1, AQP3, and AQP7 are all expressed in the jejunum, intensifying their enrollment in glycerol absorption [23]**.**

## 2 Materials and methods

### 2.1 Sample size

The sample size was calculated using G*Power software (version 3.1.9.7), according to the methods described in Ref. [24]**.** In this one-way ANOVA study, a total sample size of 16 subjects (four4 subjects per group) was obtained from the three groups whose means were to be compared. The total sample of 16 subjects achieved 97% power to detect differences between the means versus the alternative of equal means using an F test with a 0.0500 significance level. The size of the variation in the means is represented by the effect size η^2^ = σm^2^/(σm^2^ + σ^2^), which is 1.36662, as calculated using a previous study [25]**.**

### 2.2 Experimental animals

Sixteen male Sprague-Dawley albino rats (9–10 weeks old, weighing 200–250 g) were used in this study. Rats were kept in pathogen-free environments with controlled temperatures (23° ± 3 °C) and relative humidity in metal cages with soft wood chips as bedding. Animals were given conventional basal food and water for two weeks with a 12-h light-dark cycle to acclimatize them to the environment and guarantee normal development and behavior.

### 2.3 Experimental design

The rats were divided randomly into control rats (n = 4) fed a basal diet (10% fat) and water ad libitum for 12 weeks;, IF control rats (n = 4) followed the IF protocol, HFFD fed rats (n = 8) fed high fat -high fructose diet for 8 weeks, and from the beginning of the 9th week until the end of the experiment, rats were randomized into two groups high-fat high-fructose diet (HFFD) only or HFFD and IF protocol.

#### 2.3.1 Intermittent fasting (IF) protocol

Animals were fasted alternative day fasting (ADF) 24 h every other day (24 h feeding then 24 h fasting) [26]**.** On fasting days, no food was allowed, but unlimited water was available. On the feeding day in the control fasting group, the basal diet was given and, in the IF + HFFD rats were fed the HFFD protocol.

#### 2.3.2 High fat and fructose diet (HFFD)

HFFD is composed of 45% fat (40% animal fat (lard) + 5% in basal diet) [27] and fructose 20% (20 g of fructose diluted in 100 mL of tap water) [28]**.**We used lard because it is highly recommended as a standard fat to induce a valid rat model of metabolic syndrome [4]**.** The HFFD group was given food intake comparable to that measured in HFFD + IF rats to rule out the possibility of reduced calorie intake in the IF- treated group.

### 2.4 Specimens collection

All rats were weighed at the assigned times points, to estimate serum cholesterol, triglycerides, low-density lipoprotein cholesterol (LDL), and high-density lipoprotein cholesterol (HDL) levels, and blood samples were collected from the tail vein. Blood samples were allowed to coagulate at room temperature before being centrifuged, separated from the serum, and stored at 20C until analysis. The rats were rendered completely unconscious by intraperitoneal administration of xylazine (15 mg/kg) and ketamine (90 mg/kg) [29] then sacrificed. The abdomen was immediately opened through a midline incision, and the jejunum was identified and dissected, cleaned of luminal contents, and divided into three parts for subsequent analysis: the first portion was taken for a quantitative real-time polymerase chain reaction (qRT-PCR) and immersed directly in RNA Stabilization Reagent;, the second portion was taken for measuring tissue oxidative markers, malondialdehyde (MDA) and glutathione (GSH), dipped in phosphate-buffered saline kept at −80 °C; and the third portion was taken for routine histopathological examination preserved in 10% neutral buffered formalin.

### 2.5 Biochemical studies

#### 2.5.1 Lipid profile assessment

Using a biochemical analyzer (Pentra C200, Horiba, Tokyo, Japan), blood plasma levels of total cholesterol (TC), triglycerides (TGs), LDL and HDL were assessed [30]**.**

#### 2.5.2 Oxidative stress evaluation (oxidative stress markers)

In a low-temperature homogenate machine, ice-cold saline was introduced after the gut mucosa was triturated in a pre-cooled mortar. The tissue homogenate was centrifuged.

##### 2.5.2.1 MDA assay

Using the standard method, MDA, a product of lipid peroxidation, was measured in jejunal tissues. Thiobarbituric acid reactive substance (TBARS), a chromogen detected by a spectrophotometer, is produced when MDA interacts with thiobarbituric acid (Biodiagnostic, Egypt). Readings of an MDA standard, against which the following equation was used to calculate the samples:

MDA (nmol/g tissue used) = [A sample/A standard 10/g per tissue used], where A is absorbance [31]**.**

##### 2.5.2.2 GSH assessment

GSH serves as a co-substrate for glutathione peroxidase (GPx) activity and as a direct free radical scavenger. This was evaluated using the approach outlined by **Li, Xu** [32]**.**

#### 2.5.3 Quantitative real-time PCR (qRT-PCR) for AQP1, AQP3 and AQP7 mRNA

Jejunum tissue samples (50 mg) were placed in 500 μl of RNA later (Qiagen, Germany) and kept at 2–8 C for an overnight, and then at − 80 °C. Liquid nitrogen was used to homogenize tissue samples. Total cellular RNA was obtained using the TRIzol reagent (Zymo Research, Irvine, CA, USA). The concentration and purity of RNA were assessed using Nanodrop 2000 (Thermo Scientific, USA). The SensiFAST cDNA Synthesis Kit (Bioline, Memphis, TN) was used to transcribe cDNA from 1 μg of the RNA obtained with a final reaction volume of 20 μl reactions (1 μl cDNA, 4 μl 5x TransAmp Buffer, 1 μl reverse transcriptase, and 13 μl RNase-free water).

Amplification of the cDNA templates was performed using an Applied Biosystems 70500 Real-Time PCR System (Waltham, Massachusetts, USA). Ingredients for the reaction, which had a volume of 20 μl, were as follows: 1 μl of cDNA template, 10 pmol/l of gene primer, 2 μl of HERA SYBR Green PCR Master Mix (Willowfort, UK), and 7 μl of nuclease-free water. The real-time PCR thermal conditions were adjusted to 95°°C for 2 min, followed by 40 cycles of denaturation for 10 s at 95 °C, and annealing and extension for 30 s at 60 °C. The primers used for cDNA amplification are listed in Table (1). The Primer-BLAST program (NCBI/primer-BLAST) [https://www.ncbi.nlm.nih.gov/tools/primer-blast/] was used to determine primer specificity. The relative expression or fold change (RQ) of the target genes was calculated using the (2−ΔΔCt) method [33], which was normalized to the endogenous control Glyceraldehyde-3-phosphate dehydrogenase (GAPDH).

### 2.6 Histopathological assessment

The jejunal samples were opened, washed with saline, fixed in Bouin's solution for 6 h, dehydrated in alcohol, diaphanized in xylol, and embedded in paraffin. Hematoxylin-eosin (HE) staining was performed on jejunal slices that were 4 μm thick [34]**.** Using low-power digital photomicrographs (magnification 100×), intestinal histomorphometry parameters were assessed in each section: crypt depth (CD) and villous height (VH). Twenty perfectly aligned villi and matching crypts from each part of all the intestinal segments were used to quantify these characteristics. Villi's heights were specified from the tip to the base [35]**.** The depth of intestinal crypts was measured as the distance from the crypt-villus junction to the base of the crypt [36]**.**

### 2.7 Immunohistochemical assessment (AQP1, AQP3 and AQP7 immunostaining)

Prior to immunohistochemical labelling, dewaxing in xylene, rehydration, and immersion in 3% hydrogen peroxide were performed to inactivate endogenous peroxidases. sections were treated with 10 mM citrate buffer (PH 6.0) for 30 min at 95 °C. Primary polyclonal AQP1 antibody (GB11310-1, Servicebio, Wuhan, China) was incubated with sections at a 1:850 dilution, primary rabbit polyclonal antibody AQP3 (A2838 ABclonal, Woburn, MA 01801 USA at1:100 dilution), and primary rabbit polyclonal antibody AQP7 (bs-2506R, Bioss antibiotic, Woburn, MA 01801, - U.S.A., 1:100 dilution) at 4 °C overnight. Slides were treated with a secondary antibody on the day after rinsing in phosphate-buffered saline. Immunoreactivity was observed as a brown color on the slides after diaminobenzidine (DAB) staining (mMouse and rabbit HRP/DAB (ABC) detection IHC kit, ab64264, Abcam, UK). Hematoxylin was used as the counterstain.

### 2.8 Image analysis

For individual animals in all groups, five random sections using a 40X objective (area: 0.071 mm2) were examined. The uniformity of the section thickness was considered for legitimate comparisons. Light microscopy (Olympus model BX53, Tokyo, Japan) images were captured using a digital camera (Toup Cam type BX53, Japan) linked to a computer. Immune positive reactions of AQP1, AQP3, and AQP7 were analyzed using Image-J (version 1.48). We utilized the color deconvolution tool from the plugin to analyze the immunological positive response, and then used the threshold tool specified to estimate the area fraction of brown color **Heddleston**, **Aaron** [37].

### 2.9 Statistical analysis

IBM -SPSS software (Version 26) was used for data analysis. Shapiro-Wilk's test was used to determine if the data had a normal distribution because a value of P > 0.05 indicated the results. By analyzing the box plots, no notable outliers were identified. Quantitative data are presented as mean ± standard deviation (SD). All data were analyzed using one-way repeated measures ANOVA and post hoc comparison tests. Homogeneity

Of variance test was performed and the p value was non-significant (p > 0.05) so the tukey test was used. For all quantitative data, the results were deemed statistically significant at P ≤ 0.05.

## 3 Results

### 3.1 Impact of HFFD and IF on body weight

**Fig. 1:**
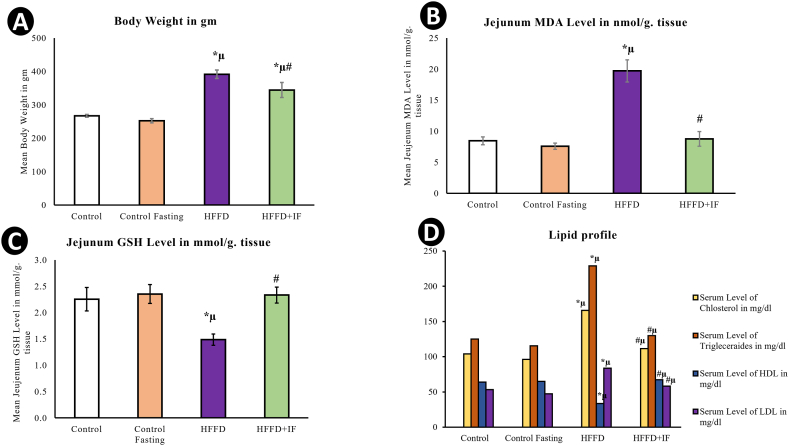
Histograms; (A) the body weight and (B) Jejunal MDA (C) Jejunal GSH level (D) Lipid profile in the experimental groups. * Significant compared to control. μ significant compared to control fasting. # significant compared to HFFD group. The results were considered significant at P < 0.05.

In comparison to the control and control fasting groups, there was a statistically significant rise (*P* < 0.05) in body weight in the HFFD group. On the other hand, HFFD treated with IF showed significant decrease (*P* < 0.05) of the body weight compared to HFFD as shown in Fig. 1A.

### 3.2 Impact of HFFD and IF on the lipid profile

Compared to the control group, the HFFD group's blood levels of TC, TG, and LDL increased significantly (*P* < 0.05), whereas HDL levels decreased significantly (*P* < 0.05). Compared with the HFFD group, the HFFD + IF had significantly lower TC, TG, and LDL levels (*P* < 0.05) and significantly higher HDL levels (*P* < 0.05) (Fig. 1D).

### 3.3 Evaluation of HFFD and IF impact on the oxidative stress markers

Comparing the HFFD group to and control group, there was a substantial increase (*P* < 0.05) in MDA and a significant drop (*P* < 0.05) in GSH levels. MDA levels were substantially lower in the HFFD + IF group than in the HFFD group (*P* < 0.05), whereas GSH levels were significantly higher (*P* < 0.05) (Fig. 1B and C). These results indicate that IF enhances the antioxidative defense mechanism.

### 3.4 Histopathological evaluation and morphometric observations of the jejunal tissue

**Fig. 2:**
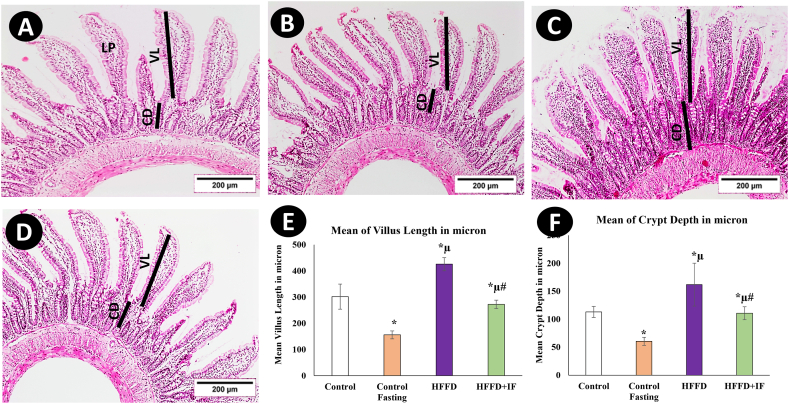
Hematoxylin & Eosin-stained sections of the rat's jejunum of control rats (A) and control fasting (B), HFFD (C), and HFFD + IF (D). In A: long cylinder villi and intestinal crypts were shown. Each villus is formed of lamina propria (LP) covered with villus epithelium composed of columnar enterocytes. Villus length (VL) and crypt depth (CD) appeared normal. E and F: Histogram of villus length and crypt depth in different experimental groups. * Significant compared to control. μ significant compared to control fasting. # significant compared to HFFD group. The results were considered significant at P < 0.05. (Magnification, × 100).

Light microscopic sections of the jejunum stained with H and E were used to examine the mucosal villi and crypts (Fig. 2 A–F). A photomicrograph of the control rat (Fig. 2A) shows long cylindrical villi and typical values of intestinal crypt depth (CD) and villus length (VL). Each villus is formed of lamina propria, covered with villus epithelium composed of columnar enterocytes. The lamina propria contained fibroblasts, plasma cells, and collagen bundles, and villus length and crypt depth were normal. Fig. 2B depicts a significant decline (*P* < 0.05) in villus length and crypt depth in the control group. HFFD caused a significant increase (*P* < 0.05) in villus length and crypt depth in compared with the control and fasting control groups (Fig. 2C). HFFD + IF showed a significant decline in villus length and crypt depth (*P* < 0.05) compared with HFFD (Fig. 2E).

### 3.5 Assessment of AQP1, AQP3, and AQP7 expression in jejunal tissue

Using qRT-PCR and immunohistochemistry, the mRNA and protein levels of AQP1, AQP3, and AQP7 were assessed in jejunal tissue samples (see Table 1).

#### 3.5.1 AQP1, AQP3 and AQP7 mRNA level

Compared to the control group, the HFFD group's AQP1, AQP3, and AQP7 gene expression (relative amount) increased considerably (*P* < 0.05). When compared to the HFFD group, the HFFD + IF had substantially lower levels of gene expression for aquaporins 1, 3, and 7 (*P* < 0.05) (Table 2).

**Table (1):** Primer sequences used for real-time PCR analysis.

Gene	Sequence	Ref Seq	Reference
GAPDH	Forward: TGGGAAGCTGGTCATCAAC Reverse: GCATCACCCCATTTGATGTT	NM_017008.4	[38]
AQP1	Forward: GCTGTCATGTATATCATCGCCCAG Reverse: AGGTCATTTCGGCCAAGTGAGT	NM_012778.2	[39]
AQP3	Forward: TGGACCTCGCCTTTTCACTG Reverse: GGAGCGTTTTTAGCCCGAGA	NM_031703.1	[40]
AQP7	Forward: GTAATGGAGGACCAGAAACAAG Reverse: TATGAGCCACGGAACCAAG	NM_019157.2	[41]

**Table 2:** The mean Relative quantity of AQP1, AQP3 and AQP7 in jejunal tissue ± SD in all studied groups.

Groups	Aquaporin 1 PCR (Mean ± SD)	Aquaporin 3 PCR (Mean ± SD)	Aquaporin 7 PCR (Mean ± SD)
Control	1.908 ±0 .053	0.988 ±0 .352	1.606 ±0 .0.051
Control Fasting	1.000 ±0 .023*	0.541 ±0 .169*	0.998 ±0 .023*
HFFD	9.671 ±0 .090*μ	2.672 ±0 .151*μ	3.646 ±0 .113*μ
HFFD + IF	2.643 ±0 .127*μ#	1.876 ±0 .267*μ#	1.768 ±0 .092*μ#

(* significance in contrast to control, μ significance in contrast to control Fasting, # significance in contrast to HFFD. P value < 0.05).

#### 3.5.2 AQP1, AQ3, and AQ7 immunoreactivity (localization)

**Fig. 3:**
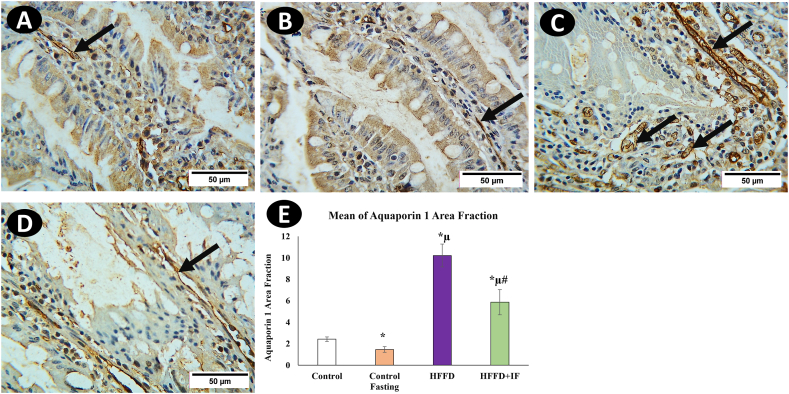
Immunohistochemical analysis for AQP1 in rat's jejunum in the control group (A), the control fasting (B), the HFFD group (C), and the HFFD + IF group (D). Arrows point to the positive reaction noticed in central lacteals. (F) Comparison of the expression between different groups. AQP1 expression was significantly increased in the HFFD group as compared to the control group. The HFFD treated with IF showed a significant AQP1 expression correlated with the HFFD group. * Significant compared to control. μ significant compared to control fasting. # significant compared to the HFFD group. The results were considered significant at P < 0.05. (Magnification, × 400).

**Fig. 4:**
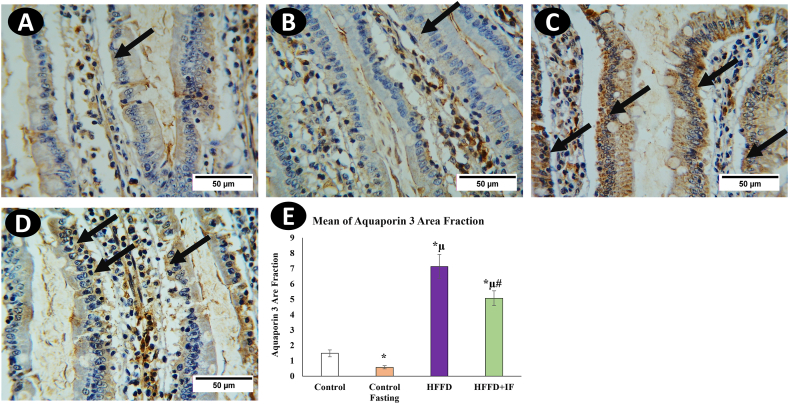
Immunohistochemical analysis for AQP3 in rat's jejunum in the control group (A), the control fasting (B), the HFFD group (C), and the HFFD + IF group (D). Arrows point to the positive reaction observed mainly in the basolateral membrane of the epithelial cells, especially those lining the upper villus part. (F) Comparison of the expression between different groups. AQP3 expression was significantly increased in the HFFD group as compared to the control group. The HFFD treated with IF showed a significant AQP3 expression correlated with the HFFD group. * Significant compared to control. μ significant compared to control fasting. # significant compared to the HFFD group. The results were considered significant at P < 0.05. (Magnification, × 400).

**Fig. 5:**
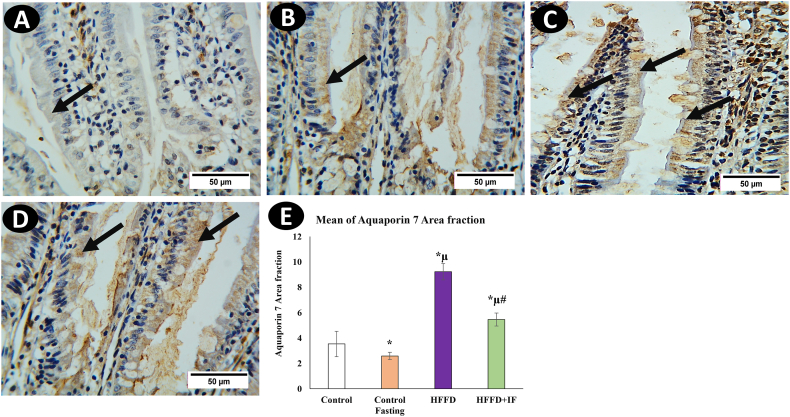
Immunohistochemical analysis for AQP7 in rat's jejunum in the control group (A), the control fasting (B), the HFFD group (C), and the HFFD + IF group (D). Arrows point to the positive reaction detected on the superficial epithelial cells of the jejunum, especially in the cells of the upper part of the villus. (F) Comparison of the expression between different groups. AQP7 expression was significantly increased in the HFFD group as compared to the control group. The HFFD treated with IF showed a significant AQP7 expression correlated with the HFFD group. * Significant compared to control. μ significant compared to control fasting. # significant compared to the HFFD group. The results were considered significant at P < 0.05. (Magnification, × 400).

AQP1 was observed in the jejunal central lacteals endothelium (Fig. 3A). AQP3 immunoreactivity was observed mainly in the basolateral membrane of enterocytes, especially those lining the upper villus, and was insignificant or even absent in the villous lower part and crypts (Fig. 4A). However, this was not observed in the crypts. AQP7 immunoreactivity was detected in superficial epithelial cells of the jejunum, particularly in the upper section of the villus (Fig. 5A). Even though intracellular staining was visible, at high magnification, AQP7 expression was stronger in the brush border membranes of epithelial cells. The basolateral membranes of enterocytes showed no staining.

#### 3.5.3 Morphometric analysis of the immunoreactive area % for AQP1, AQP3, and AQP7

Fig. 3, Fig. 4, Fig. 5E illustrate the immune immunoreactive area fractions of aquaporin's 1, 3, and 7, respectively, in the different studied groups. The immune expressions of aquaporin's 1, 3, and 7 in the HFFD group were significantly higher than those in the control group. In the IF-treated HFFD group, aquaporin 1, 3, and 7 immune expressions were significantly decreased in comparison with the HFFD group. It was observed that the intermittent fasting control group showed a significant decrease in aquaporin 1 (Fig. 3B–D), 3 (Fig. 4B–D) and 7 (Fig. 5B–D) immune expressions compared to the control group.

## 4 Discussion

Consumption of saturated fat and simple carbohydrates, particularly fructose, has grown considerably over the past century owing to their inclusion in processed foods such as fast food, jellies, and soft drinks [7]. This consumption affects the intestines by altering the ratio of barrier-forming cytokines to barrier-disrupting cytokines [8]. Aquaglyceroporins permit glycerol to pass through intestinal membranes, which is a prerequisite for maintaining energy homeostasis and regulating fat synthesis [11].

The focus of this study was to assess alterations in AQP1, AQP3, and AQP7 after a high-fat diet and the impact of intermittent fasting on these changes. We evaluated the parallel lipid profile changes, oxidative stress, and jejunal histopathological changes under both conditions. intestine. The intestine has received our particular attention in this research as it is the first step in the long lipid-handling process. We wanted to focus on the role of aquaporins in the first step of lipids entering the body and how it is affected by high fat and IF. Numerous studies have been conducted on subsequent steps occurring in the liver and adipose tissue. Our target was to evaluate another important new research target affecting lipids absorption. Our subsequent research on this point. It will be considered to evaluate the entire pathway of lipids and how it is affected by different aquaporins.

According to established standards, HFFD consumption causes obesity and obesity-related complications, including hyperlipidemia, resulting in greater levels of TC, TG, and LDL, and decreased levels of HDL compared to the control groups [42,43]**.** VLDL is generated in response to a large inflow of free fatty acids into the liver, which aids in the development of dyslipidemia [44]. In addition, HFFD increased the concentration of chylomicrons in the intestines. The liver absorbs the free fatty acids that are produced when these chylomicrons circulate in the bloodstream. These hepatic-free fatty acids can either be esterified into triglycerides or reach the mitochondria for B-oxidation. Triglycerides either produce VLDL, which is then transformed into LDL, or aggregates as tiny droplets in hepatocytes [45]**.**

In the HFFD group treated with intermittent fasting, there was a significant decline in TC, TG, and LDL and a significant decrease in comparison to the HFFD group, which agrees with [[46], [47], [48]]**.** The ameliorative effect of IF on the lipid profile can be explained by the hepatic modulation of several molecules. Peroxisome proliferator-activated receptor alpha (PPARα) and peroxisome proliferator-activated receptor-gamma coactivator 1-alpha (PGC-1α) promote fatty acid oxidation, apolipoprotein A production, and apolipoprotein B decline. The reduction in hepatic triglycerides, consequent to increased fatty acid oxidation, lowers the blood levels of VLDL and LDL. An increase in apolipoprotein A synthesis results in higher HDL levels. Reduced amounts of VLDL, and LDL lead to the loss of transported triglycerides and cholesterol in the body by intermittent fasting, which is reflected in a decrease in blood cholesterol and triglycerides [49]**.**

In the current study, MDA and GSH levels were evaluated in jejunal tissue; MDA level elevation and GSH level decline were observed in the high-fat diet group, in agreement with [50,51] respectively. GSH is an intracellular thiol that eliminates free radicals andor lowers hydrogen peroxide levels under oxidative stress conditions. Polyunsaturated fatty acids are oxidized by free radicals and reactive oxygen species (ROS). MDA is a biological marker frequently used to evaluate oxidative stress, as it develops as a result of the degradation of oxidized lipid molecules [52,53]**.** It is generally recognized that fatty acids raise the load of oxidative stress caused by an imbalance between ROS production and the body's antioxidant defense [54]. Excessive consumption of fat initiates mitochondrial -oxidation of free fatty acids, which in turn causes an increase in cytochrome *c* oxidase activity and ROS production [55]**.** The decrease in GSH levels may be due to its increased consumption to neutralize the increased formation of lipids peroxides [56]. In the group treated with IF, there was a decrease in the MDA level in agreement with [57] and an enhancement in GSH production in accordance with other reports [58]**.** According to Al-Shafei [53], an adaptive stress response occurs in cells in response to IF, which boosts the production of antioxidant defenses including blood glutathione, superoxide dismutase 1, and catalase.

Villus length and crypt depth were evaluated to counteract the impact of HFFD and fasting on jejunal architecture. Increases in villus length and crypt depth were observed in the HFFD group compared to the control animals, supporting the idea established by other studies [[59], [60], [61], [62], [63]] that the hypertrophic effect seen in the villi and crypts of the rat jejunum was caused by the increased digestion and absorption surface stimulated by HFFD and that the type of food consumed was the main trigger rather than the quantity. However, some studies adopted the opposite idea that villous length decreases with a high-fat diet and increases with IF [64]. This discrepancy might be explained by the different species used and different durations adopted by each research. A high-fat diet may cause the intestines to adapt quickly in certain situations; after just one day of high-fat food consumption, lipid buildup was seen along the length of the villus [65].

Our investigation showed, for the first time, to our knowledge, that IF improved the intestinal morphological changes linked to HFFD. Furthermore, as intestinal mucosal villi at the apical surface of epithelial cells promote intestinal absorptive surface area, noticeably reversed villi and crypt lengths following IF, implying a decline in intestinal absorption capacity.

This is the first study that we are aware of that compares AQP-1, AQP-3, and AQP-7 in rat jejunum to HFFD and IF. Aquaporin 1 was observed in the endothelial cells of the central lacteal jejunum, in accordance with [15]**.** AQP3 was detected in the basolateral membrane of enterocytes, whereas AQP7 was found in the apical membrane of the epithelial cells. in line with [10]**.** A significant increase in AQP1, AQP3, and AQP7 expression was observed in the HFFD group as compared to that in the control group, coinciding with the hypertrophic effect seen in rat jejunum villi and crypts stimulated by HFFD. Therefore, increasing AQP1, AQP3, and AQP7 expression could be explained by increased digestion and absorption surfaces. In contrast, AQP1, AQP3, and AQP7 expressions were considerably lower in the HFFD + IF group than in the HFFD group. These findings highlight the importance of modulating AQP1, AQP3, and AQP7 in fat absorption and the ameliorative effect of IF, which could be due to the control of these aquaporins.

## 5 Conclusion

Particular attention to the AQPs 1,3 and 7 expression modulation was an outcome of this study. Additional research is required to identify the precise processes and signaling pathways involved in the regulation of intestinal glycerol absorption and excretion. The suppression of AQPs may be an attractive anti-obesity treatment. Furthermore, IF has been shown to suppress oxidative stress and countercurrent intestinal histopathological changes by modulating AQP1, AQP3, and AQP7 expressions.

## 6 Recommendation

The specific mechanisms and individual avenues by which these AQPs regulate intestinal glycerol absorption and excretion must be determined to determine how they might be exploited as therapeutic targets.

## Contribution to the field

The widespread use of high-fat diet negatively affects personal health and causes obesity, metabolic syndrome, and other deleterious complications. Intermittent fasting appeared to be a reliable solution for controlling this problem. A transmembrane water and glycerol channel called aquaporin may be able to address this problem.

## Funding

This research did not receive any specific grants from funding agencies in the public, commercial, or not-for-profit sectors.

## Limitation of the study methodology and result application

No limitation present regarding this research.

## Data availability

Data will be made available on request.

## Ethical statement

This animal experiment complied with the ARRIVE guidelines. This stu) and approved by the Animal Care and Use Committee of Mansoura University (approval no. 22.11.32) and performed in accordance with the National Institutes of Health Guide for the Care and Use of Laboratory Animals (NIH Publications No. 8023, revised 1978).

## CRediT authorship contribution statement

**Heba M. Elhessy:** Writing – review & editing, Writing – original draft, Methodology, Investigation, Data curation, Conceptualization. **Mohamed Berika:** Writing – review & editing, Validation, Project administration, Formal analysis. **Yassmin G. Salem:** Writing – review & editing, Resources, Data curation. **Manal M. El-Desoky:** Writing – original draft, Resources, Methodology. **Mamdouh Eldesoqui:** Writing – review & editing, Writing – original draft, Investigation, Formal analysis. **Nora Mostafa:** Writing – original draft, Resources, Data curation. **Ola A. Habotta:** Project administration, Methodology, Data curation. **Nermeen H. Lashine:** Writing – review & editing, Writing – original draft, Software, Formal analysis.

## Declaration of competing interest

The authors declare that they have no known competing financial interests or personal relationships that could have appeared to influence the work reported in this paper.

The authors declare that there are no financial interests or relationships — within the last 3 years — related to the subject matter.

The authors declare that there are no patents or copyrights that are relevant to the work in the manuscript.
